# Crack propagation analysis and fatigue life assessment of high-strength bolts based on fracture mechanics

**DOI:** 10.1038/s41598-023-41804-z

**Published:** 2023-09-04

**Authors:** Ping Zhang, Jiachun Li, Yu Zhao, Jiaxiao Li

**Affiliations:** 1 College of Mechanical Engineering, Guizhou University, Guiyang, 550000 China; 2 College of Physics and Electromechanical Engineering, Guizhou Minzu University, Guiyang, 550000 China; 3 China Aviation Industry Standard Parts Manufacturing Co. LID, Guiyang, 550000 China

**Keywords:** Engineering, Materials science, Mathematics and computing

## Abstract

To investigate the effect of initial cracks on the fatigue performance of high-strength bolts for high-speed train brake discs, the fatigue crack propagation behavior of high-strength bolts under the coupling action of preload and dynamic fatigue load was investigated experimentally and numerically based on the theory of linear elastic fracture mechanics. Firstly, fatigue tests of high-strength bolts with initial crack defects were carried out, and then a three-dimensional accurate numerical model with the hexahedral mesh for a bolt-nut was established by MATLAB, and the fatigue crack propagation behaviors were investigated using ABAQUS-FRANC3D interactive technology. In this paper, the effects of the initial crack state, the bolt preload, the axial excitation load, and the friction coefficient of the screw pair on crack propagation life were emphatically studied, and the simulated crack propagation trajectory and crack propagation life agreed well with the experimental results. The findings indicated that 0°-oriented cracks beginning at the maximum principal stress were predicted to have the shortest fatigue life. The crack propagation life was sensitive to the initial crack size, the coefficient of initial crack geometry, and the bolt preload, but not to the friction coefficient of the screw pair. Furthermore, when evaluating the effect of fatigue load on crack propagation, the load ratio, the mean load, and the load range should all be considered.

## Introduction

The reliability of large-size, high-strength, and highly fatigue-resistant bolts for high-speed train brake discs is very important to the safety of train operation. Research on the fatigue property of bolts is the key to ensuring the service life of bolts, and it is of great significance to the design and processing of bolts. The effect of dynamic load on train operation can be counteracted by the effectiveness and safety of high-strength bolted connections^1,2^. However, more than 70% of engineering accidents are caused by fatigue failure of high-strength bolts during normal service^3^. This is due to the variabilities of the geometry and load transfer^4^, stress is concentrated at the thread bottom for the majority of bolts^5^. In the case of cyclic loading and a harsh environment, the crack will be initiated at the root of the thread after repeated cycles for the sake of fatigue. With the circulation of external loads, the crack will be expanded, and finally, the fracture of the bolt will be caused, resulting in the occurrence of serious accidents. Therefore, a thorough understanding of the fatigue behavior of bolts and their sensitivity to the initial crack state, load circumstances, and the friction coefficient of the thread pair can provide valuable recommendations for optimizing the design of bolts to prolong service life and improve fatigue performance.

For most fatigue failures, the usual experimental assessments are cost-efficient and time-consuming; therefore, numerical simulation techniques will probably be better suited to researching the fatigue crack propagation of high-strength bolts. At present, to simulate fatigue fracture, different numerical methods such as the finite element method (FEM)^6,7^, the boundary element method^8^, the mesh-less method^9^, and the extended finite element method (XFEM)^10^ have been utilized.

However, the crack propagation of bolts can’t be accurately simulated by traditional methods due to the influence of the complex three-dimensional geometry. Based on the M-integral, a more representative SIF solution is provided by FRANC3D to assess crack propagation life, crack propagation path, fracture, and fatigue life, which has been proven very successful in many respects^11–14^.

The fatigue fracture process of high-strength bolts is generally separated into three stages, namely, crack initiation at the bottom of the thread, stable crack propagation along the root of the thread, and rapid fracture stage, and the first two stages can basically be considered as the entire bolt life cycle. At present, the fatigue research on bolts is mostly focused on the whole fatigue life cycle and the fatigue crack initiation stage. By constant amplitude fatigue test, the effects of stress ratio, stress amplitude, pre-tightening force, and bolt grade on the fatigue properties of high-strength bolts have been studied by Xu Yazhou^15^, Shen Yu^16^, Yang^17^, Jiao^18^, and Maljaars^19^. In their research, the macro-morphology of bolt fracture was analyzed, the S–N curve was fitted, and the fatigue life formula was given. By producing a series of S–N curves for different levels of preload, the effect of preload on the fatigue life of bolts was investigated by Shahani ^20^. Ajaei^21^ studied the influence of insufficient preload on the fatigue demand of wind turbine tower bolts and concluded that the decrease in bolt preload leads to an increase in fatigue damage. Lei Honggang^22,23^ conducted intensive research into high-strength bolt failure mechanisms under varied amplitude fatigue, and the calculation method for the bolt fatigue life was obtained. Research on the crack propagation life of high-strength bolts is seldom reported. However, the fatigue crack propagation life of high-strength bolts is a crucial aspect of their entire life cycle. Therefore, it is very important to research the fatigue crack propagation of high-strength bolts.

Due to the complicated coupling effects of stress concentration and dynamic fatigue, a variety of factors can influence the formation of fatigue crack propagation in high-strength bolts^24^. Nevertheless, in the fatigue crack propagation problem, the main factor determining the path and rate of crack propagation is the stress field around the crack tip, namely the stress intensity factor (SIF). Through experimental research and numerical simulation, Saber^25^ studied the influence of the size, location, and geometry of the notch on the AISI 1045 steel plate on the fatigue life and crack propagation path, Reza Masoudi Nejad^26^ studied the fatigue crack propagation behavior and optimized the fatigue life of riveted joints in Al-alloy 2024 plates. These studies all showed that the numerical results were in good agreement with the experimental results, and the effectiveness of the research methods was verified. Thus, the establishment of an accurate fatigue crack propagation model is a key tool for estimating the fatigue life of bolts. In this study, the fatigue crack propagation behavior of high-strength bolts under the coupling action of preload and axial cyclic loading was investigated by experimental and computational methods based on the theory of linear elastic fracture mechanics. 3D crack propagation was evaluated by the interactive simulation of finite element software ABAQUS and FRANC3D, and the influence of the key variables on the growth of bolt fatigue cracks was analyzed in depth. The accuracy of the predicted outcomes was confirmed by the corresponding experiments, which provide important guidance for the optimal design of high-strength bolts.

## Fatigue crack propagation theory

### The calculation of the Stress intensity factor

#### Equivalent stress intensity factor

There are three possible modes for the stress field around a crack tip when the fracture crack develops in an isotropic homogeneous material: Mode I (opening), Mode II (sliding), and Mode III (tearing). The Stress Intensity Factor (SIF) is used to describe the stress field near the crack tip and to create fracture criteria to determine whether or not the linear elastic materials experience rapid and unstable fatigue crack propagation^27^. Specifically,

1 $$ K_{equiv} = \frac{{K_{I} }}{2} + \frac{1}{2}\sqrt {K_{I}^{2} + 4\left( {1.155K_{II} } \right)^{2} + 4K_{III}^{2} } $$

where $$K_{equiv} { }$$ is the equivalent of SIF, $$K_{I} ,\;K_{II}$$ and $$K_{III}$$ are the stress intensity factors for modes I, II, and III, respectively. According to the associated theories of fracture mechanics^28,29^, when $$K_{equiv} $$  ≥ $$ K_{C}$$, where $$K_{C}$$ is the critical SIF of the bolt material, the crack propagation enters the rapid instability stage.

#### M-integral

The M-integral, i.e., the interaction integral, is applied to linear elastic fracture mechanics, which is a crack path-independent technique based on the J-integral for calculating the global energy release rate to determine the stress intensity factors $K_{I} ,\;K_{II}$ and $K_{III}$ of the three fracture modes. The equivalent domain equation of M-integral is the most accurate method for calculating the Stress intensity factor, and the effect of additional factors, such as temperature, residual stress, the friction factor of the crack surface, etc., can be considered^30^, namely,

2 $$ M = \oint_{\Gamma } {\left( {Wx_{i} n_{i} - T_{j} u_{j,i} x_{i} } \right)} ds $$

where г is an arbitrary integral path around the crack tip, $$W$$ is the density of strain energy, defined as $$ W = \frac{1}{2}\sigma_{ij} \varepsilon_{ij}$$, $${\upsigma }_{ij}$$ is the stress tensor, $${{\varvec{\upvarepsilon}}}_{{{\varvec{ij}}}} { }$$ is the strain tensor, $$n_{i}$$ is the directional cosine of the exterior normal for the path г, $$T_{j} $$ is the surface force vector outside the integral path, $$u_{j,i}$$ is the displacement vector on the path г, $$x_{i}$$ is the plane coordinate system, $$s$$ is the integral arc microelement.

### Sub-critical crack propagation

The sub-critical crack propagation includes the calculation of the local node kink angle $\Delta \theta_{c}$ and local node propagation $\Delta a_{i}$ along the crack front, and then the crack front is fitted and extended.

#### Kink angle model

During the growth of a crack, the growth direction is represented by the kink angle $\Delta \theta_{c}$. As a way to help estimate the direction in which cracks will propagate, many criteria, including the maximum tangential stress (MTS) criterion, the maximum shear stress (MSS) criterion, the strain energy release rate (SERR) criterion, etc., have been developed. Among them, the maximum circumferential stress criterion is more intuitive and has been verified by many experimental observations, so it has been widely concerned and applied. This method is also used to determine the crack propagation angle in this paper.

**Figure 1:**
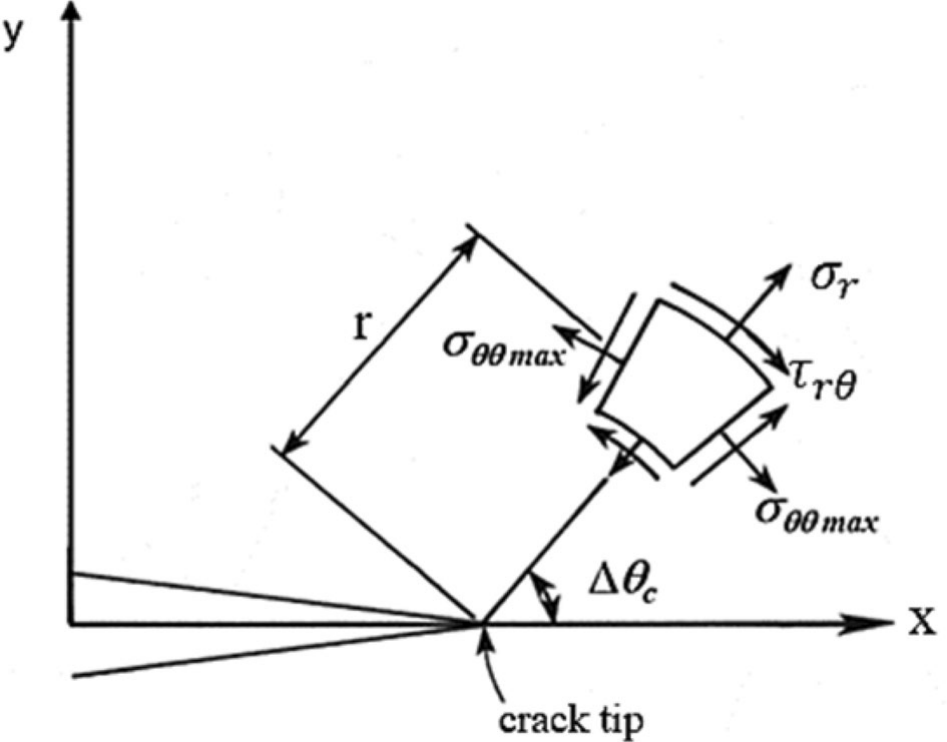
Maximum circumferential stress (MTS) criterion.

The principle of the MTS criterion is shown in Fig. 1: SIF is calculated at each node of the crack front to determine the kinking angle $\Delta \theta_{c}$, and the crack will be kinked in the direction perpendicular to the maximum circumferential stress.

By using linear elastic fracture mechanics^31^, the circumferential stress in polar coordinates is determined to be

3 $$ \sigma_{\theta \theta } = \frac{1}{{\sqrt {2\pi r} }}\cos \frac{\theta }{2}\left[ {K_{I} \cos^{2} \frac{\theta }{2} - \frac{3}{2}K_{II} \sin \theta } \right] $$

where (r, θ) is the local polar coordinate system with the origin centered at the crack tip.

When a crack propagates along the direction of maximum circumferential stress^32^, the circumferential stress intensity factor $K_{\theta \theta }$ is expressed as

4 $$ K_{\theta \theta } = \mathop {Lim}\limits_{r \to 0} \sigma_{\theta \theta } \sqrt {2\pi r} = \cos \frac{\theta }{2}\left[ {K_{I} \cos^{2} \frac{\theta }{2} - \frac{3}{2}K_{II} \sin \theta } \right] $$

Thus, the circumferential stress ${\upsigma }_{\theta \theta } { }$ of the crack can be rewritten as

5 $$ \sigma_{\theta \theta } = \frac{1}{{\sqrt {2\pi r} }}K_{\theta \theta } $$

According to this criterion, the direction of crack propagation $\theta_{c}$ at each crack tip is obtained by setting the local shear stress to zero, namely,

6 $$ K_{I} \sin \theta_{c} \pm K_{II} (3\cos \theta_{c} - 1) = 0 $$

In this case, the crack propagation angle is

7 $$ \Delta \theta_{c} = 2\tan^{ - 1} \left\{ {\frac{{1 - \sqrt {1 + 8\left( {K_{II} /K_{I} } \right)^{2} } }}{{4\left( {K_{II} /K_{I} } \right)}}} \right\} $$

#### The extension $\Delta a_{{\varvec{i}}}$

**Figure 2:**
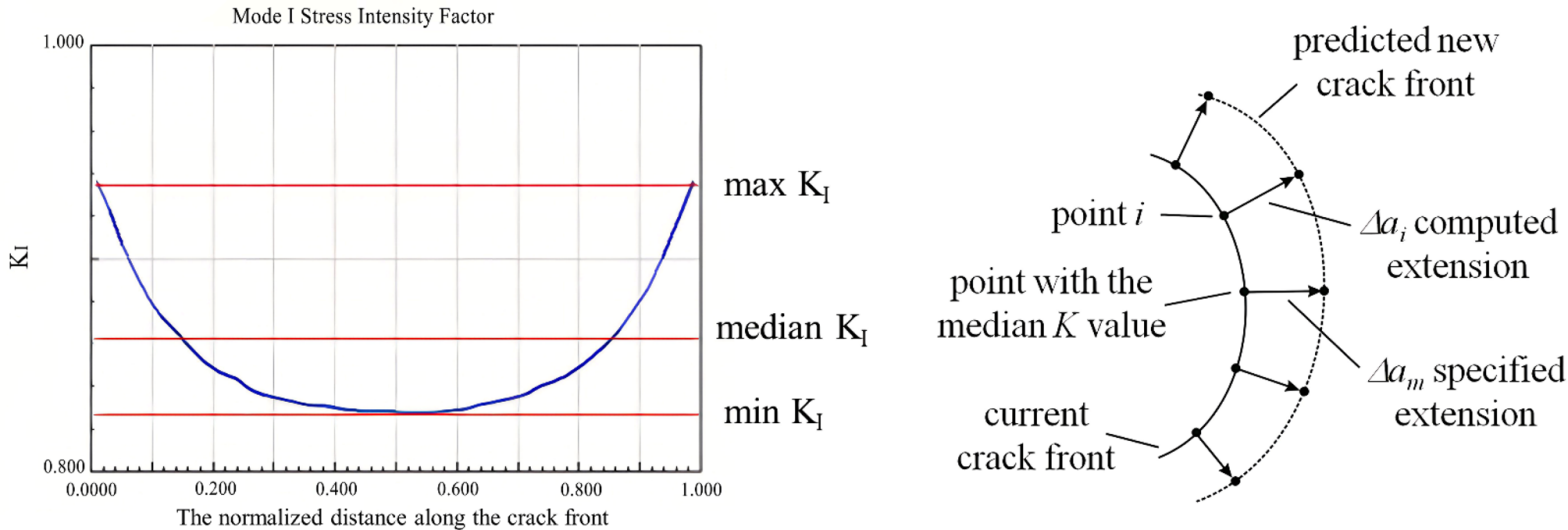
Mode of crack front propagation.

Two extension types are provided for fatigue crack propagation in FRANC3D: median extension and cycle extension. In this paper, the median extension was adopted, in which the extension at the node on the crack front is specified and the node represents the stress intensity factor mean (median K) of the crack front, as shown in Fig. 2.

If the extension of the node at the median of the crack front is defined as $\Delta a_{m}$, the extension at other nodes is expressed as:

8 $$ \Delta a_{i} = \Delta a_{m} \times \left( {\frac{{\Delta K_{i} }}{{\Delta K_{median} }}} \right)^{n} $$

where n is the rate parameter m of crack propagation in the Paris formula.

**Figure 3:**
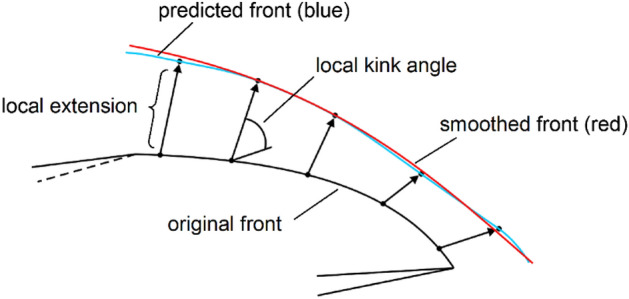
SIF is used to predict the propagation direction and relative distance of the crack front.

When fatigue crack propagation is simulated by using the mean value theorem, the nodes at the original crack front shift to new places after utilizing the fatigue growth model to calculate the results. These nodes can be connected at new locations to create a fresh crack front. Because it immediately connects many sites, the new crack front is often serrated frequently. Therefore, a third-order polynomial spline is needed for smoothing, as shown in Fig. 3.

### Crack propagation rate model

In regions with stable expansion, the crack propagation rate is frequently calculated using the Paris equation, but it is not applicable to regions of initial crack expansion and final rapid expansion, since the crack expansions of both stages are nar^28^. To estimate the rate of crack propagation across the entire crack propagation stage, the crack propagation rate model of NASGRO was adopted, which represents an upgrade to the FORMAN model I. In this model, the magnitude of the stress intensity factor is the key component influencing crack propagation, with the impacts of the stress ratio, the propagation threshold, and the fracture toughness all being taken into account^33^.

9 $$ \frac{da}{{dN}} = C\left[ {\left( {\frac{1 - f}{{1 - R}}} \right)\Delta K} \right]^{n} \frac{{\left( {1 - \frac{{\Delta K_{th} }}{\Delta K}} \right)^{p} }}{{\left( {1 - \frac{{K_{max} }}{{K_{c} }}} \right)^{q} }} $$

where $$\frac{da}{{dN}}$$ stands for the rate of crack propagation, $$ f$$ is a function of crack opening, $$R$$ is the load ratio, $$\Delta K$$ is the range of SIF, c, $$n$$, $$p$$ and $$q$$ are material parameters, $$\Delta K_{th}$$ is the threshold of SIF amplitude, $$K_{max}$$ is the maximum SIF, and $$K_{c}$$ is the critical SIF.

According to the material library provided by FRANC3D, the parameters of the tested material, A286, are shown in Table 1.

**Table 1:** Material properties as used in the NASGRO version 3 equation (Eq. 9) for A286.

Material	KC(MPa.$\sqrt {{\text{mm}}}$)	C(mm/cycle)	n	p	q
A286	4865	4.4257e-11	2.1	0.25	0.25

## Fatigue crack propagation test and the validation of the simulation method

The purposes of the fatigue tests were to investigate the process of fatigue crack propagation, acquire the fatigue crack propagation sections of high-strength bolts, and then verify the correctness of the fracture model and calculation method by contrasting the test findings with the simulation results.

### Fatigue crack propagation test

**Figure 4:**
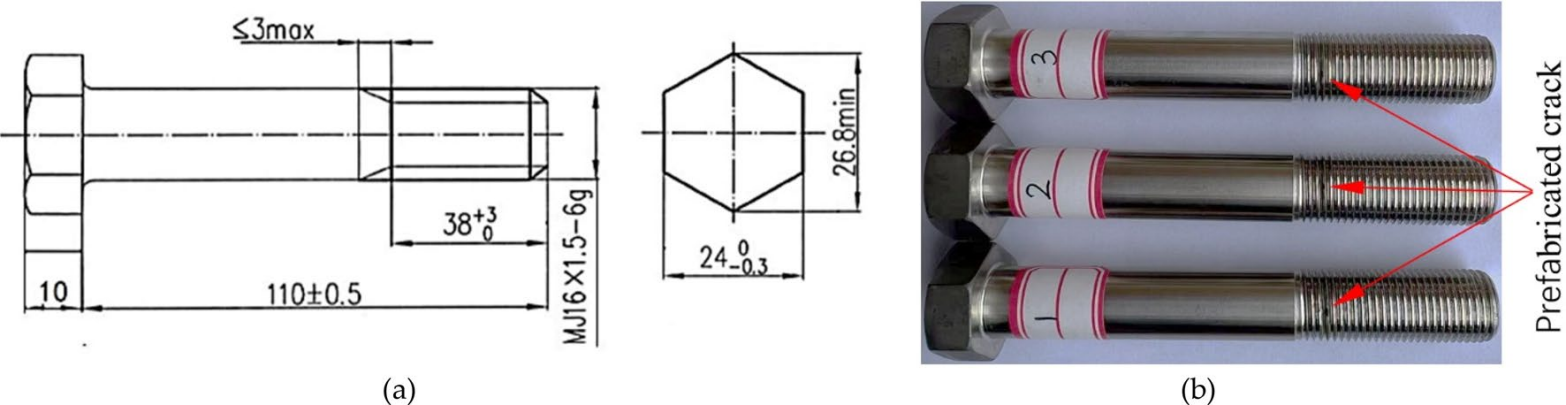
Experimental samples: (**a**) 2D structure; (**b**) test samples.

In the experiment, the test samples were hexagonal head high-strength bolts for high-speed train brake discs. The 2D structure and the test samples are shown in Fig. 4. Technical specifications include M16 × 1.5, a tooth angle of 60°, a total length of 120 mm, and an effective length of 38 mm of screw thread. The material of the bolts is A286, Fe-25Ni-15Cr, a Fe-based superalloy, which has good processing plasticity and thermal stability. Through a static tension test according to China Aviation Industry Standard Parts Manufacturing Co. LID, the modulus of elasticity is 201GPa, the Poisson's ratio is 0.306, the yield strength YS is 1310.05 MPa, and the tensile strength TS is 1379 MPa.

**Figure 5:**
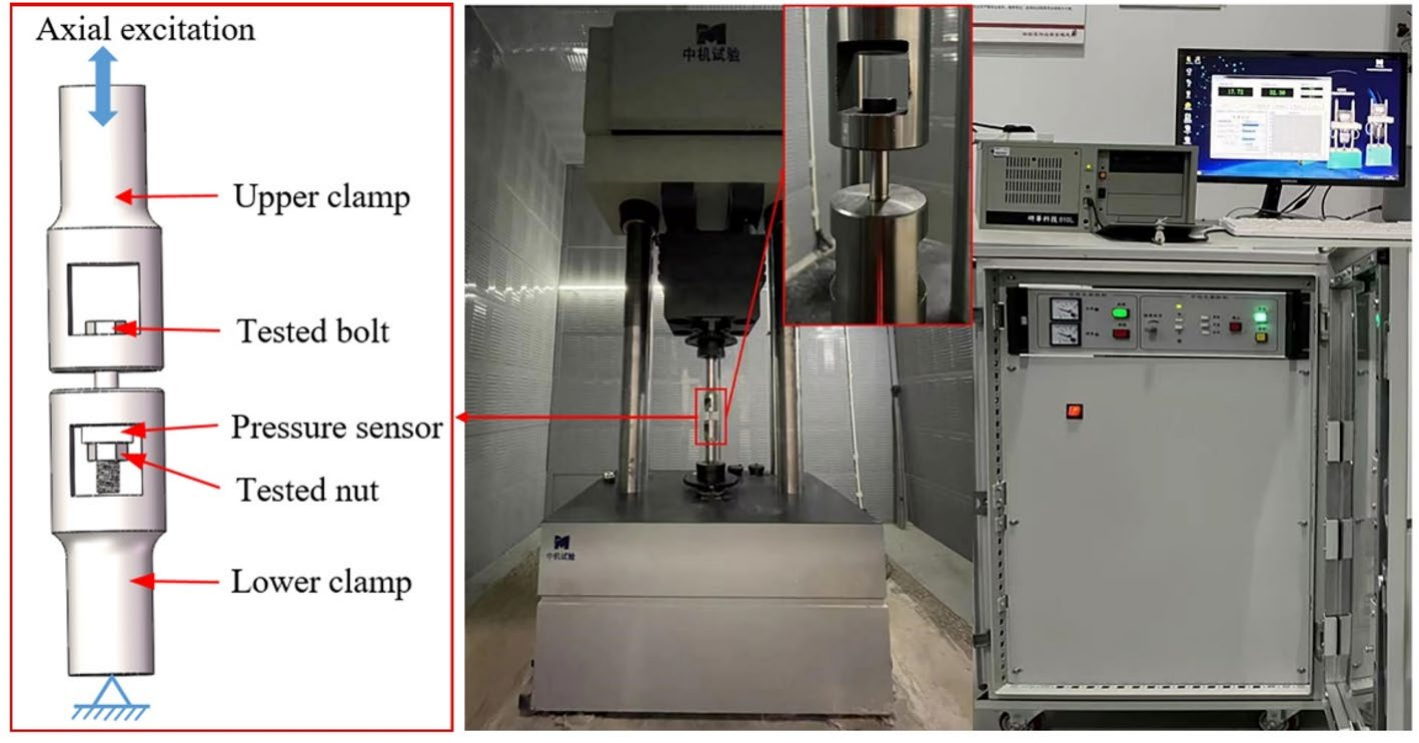
QGB-500 electromagnetic resonance fatigue testing machine.

An elliptical micro-crack with a depth of 0.5 mm was prefabricated by using wire-cut machining with a diameter of 0.08 mm at the root of the first engagement thread in the test bolt, as shown in Fig. 4b. The tension-tension constant amplitude fatigue life test was carried out to obtain the fatigue crack propagation life of the bolts on the QGB-500 electromagnetic resonance fatigue testing machine at room temperature by simulating the coupling action of preload and axial alternating load under the actual working state. The experimental equipment is shown in Fig. 5.

Due to the influence of plane strain, the fatigue crack propagation rate inside the bolt is generally faster than that of the surface crack. It is difficult to obtain the fatigue crack propagation dimensions directly by the existing methods.

**Figure 6:**
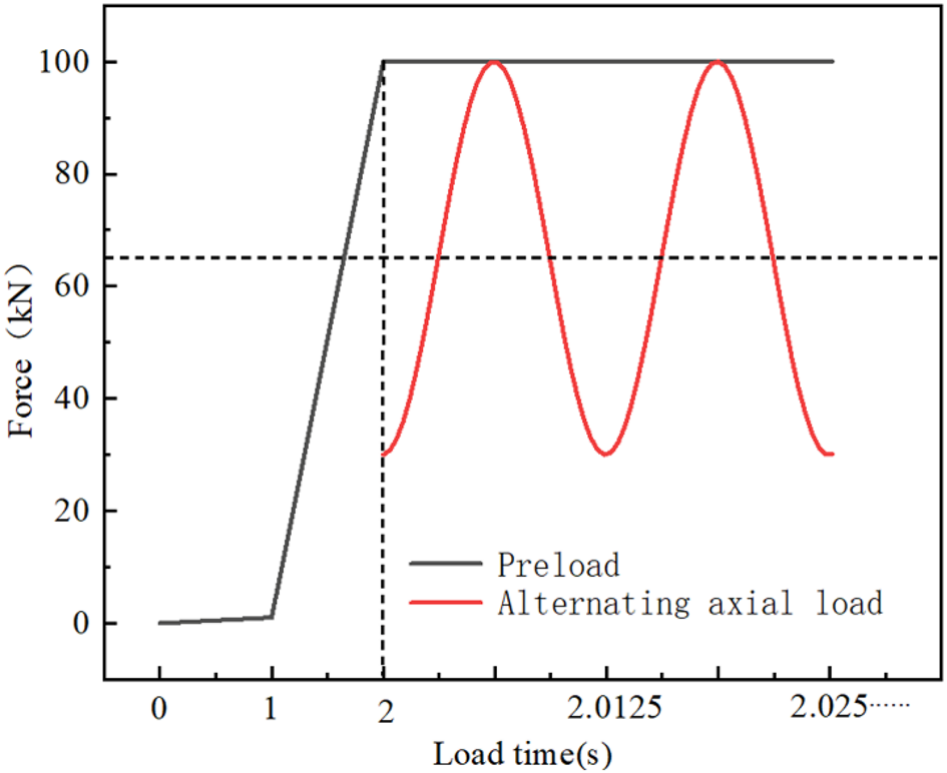
Loading process.

The cyclic load was transmitted to the bolt by the testing machine through the machine clamp: the lower clamp was fixed and the upper clamp moved slightly up and down. According to the pre-tightening force setting standard for the high-strength bolt and its application occasion, the tension-tension fatigue test was carried out under constant amplitude excitation with a load frequency of 80 Hz, a preload of 100 kN, a maximum axial load of 100 kN and a load ratio of 0.3. The loading process is shown in Fig. 6.

### Numerical simulation

#### Modelling in ABAQUS

Insufficient mesh accuracy arose from the direct utilization of ABAQUS for meshing due to the intricate nature of the thread structure. In this paper, MATLAB programming was used to calculate the node coordinates and element numbers of the threaded part. Then, these data were imported into ABAQUS to generate a three-dimensional hexahedral mesh model of the thread teeth. The modeling of the screw part was completed directly in the ABAQUS software. Finally, an accurate 3D finite element model of a bolt-nut fatigue test assembly without preset cracks was established by a Boolean operation. For the uncracked model, the C3D8R unit type was used by all components, with a total of 400,721 nodes and 366,904 elements, and the minimum element size is 0.13 mm.

Three contact pairs were defined in the finite element model: the contact between the bolt head and the upper clamp (contact 1), the contact between the nut and the lower clamp (contact 2), and the contact between the bolt and the nut (contact 3). According to the actual load condition, the finite sliding was set to each contact pair, and the tangential and normal behaviors were simulated by the penalty function method with a friction coefficient of 0.15 and the hard contact, respectively.

**Figure 7:**
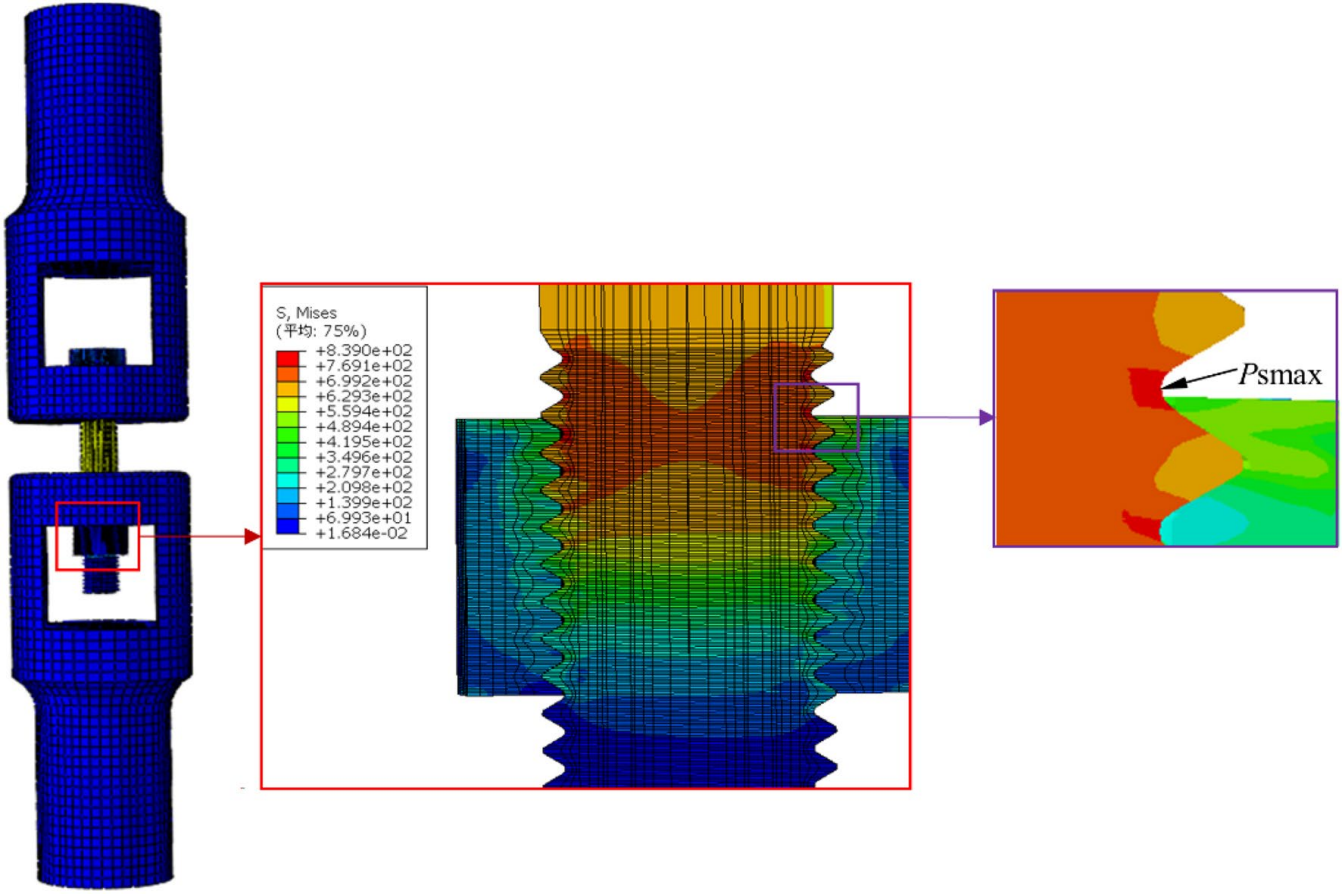
The stress distribution under the action of pretension.

**Figure 8:**
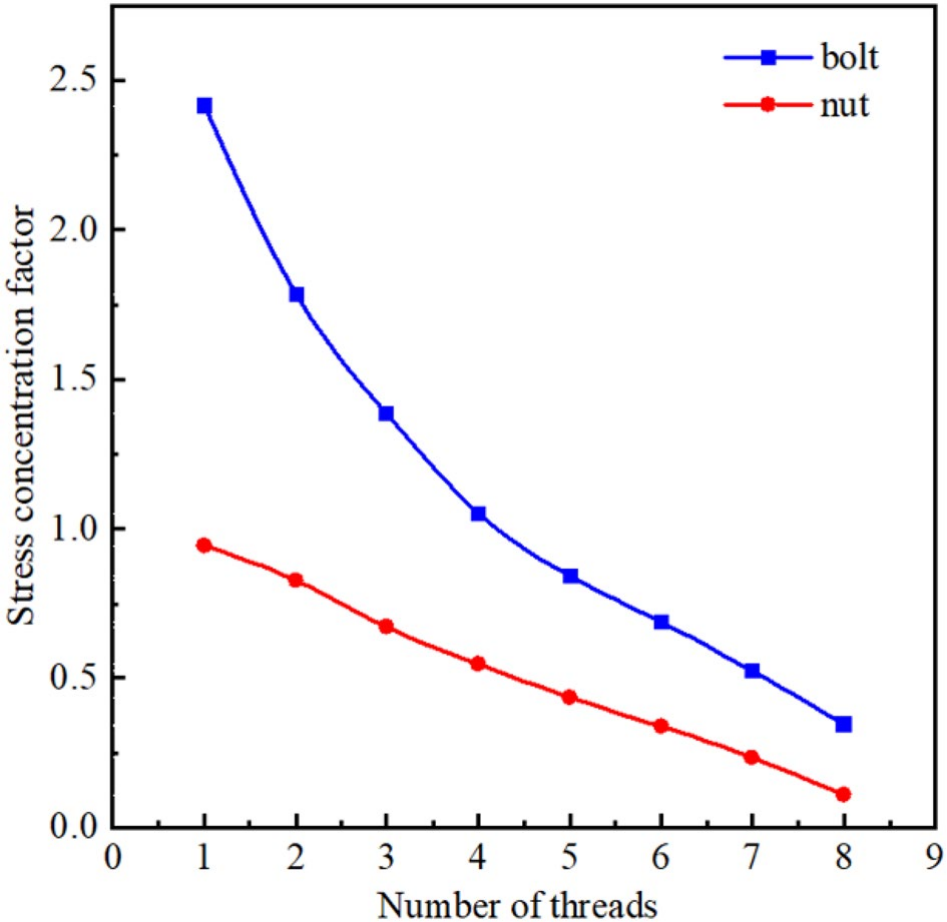
The stress concentration factor under the action of preload.

Then, the stress distribution of the bolt-nut connector under the preload was analyzed using Abaqus^34–36^. Following the experimental conditions described in 3.1, the material properties and boundary conditions were set, and the angle of rotation was applied to the nut to simulate the pre-tightening force acting on the bolt for predicting the magnitude and location of the maximum stress. The simulation result is shown in Fig. 7. Based on the simulation results, the stress concentration coefficient of each tooth root in the engagement section of the bolt-nut was calculated. As shown in Fig. 8, the bolt has the most severe stress concentration at the tooth root of the first engagement button with the nut, where the plastic deformation is the greatest. Therefore, the bolts are easy to generate fatigue cracks and fatigue fractures under the action of the axial alternating load on the upper end of clamp 1, which is in good agreement with the experimental conditions.

#### Modelling in FRANC3D

**Figure 9:**
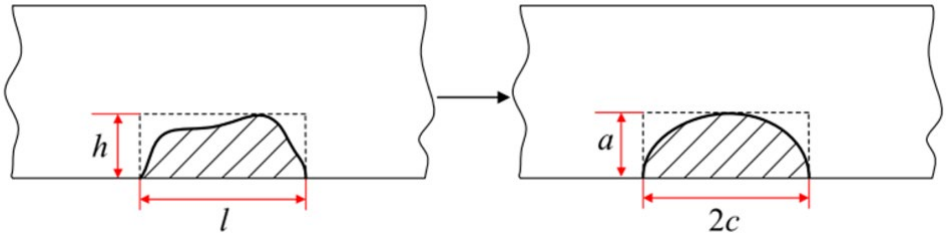
Standardization of initial crack shape.

Based on the interaction of FRANC3D and Abaqus, the initial crack was presupposed to occur at the maximum stress point of the bolt. In order to simulate the fatigue crack propagation accurately, the initial size and shape of the three-dimensional crack must be determined firstly. The actual initial crack leading edge is usually an irregular curve, which is difficult to be determined experimentally. Therefore, the length of the crack front needs to be normalized. The finite element simulation of the Dhamari study shows that any initial crack shape will rapidly develop into a common profile, after which, the crack will propagate in a similar manner^37,38^, as shown in Fig. 9. Common surface cracks can be simplified, where the crack length is 2c and the crack depth is a. A simple semicircular initial crack shape is postulated in this research.

**Figure 10:**
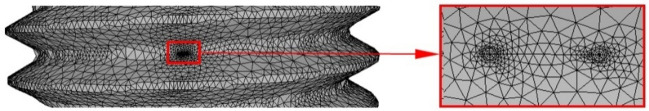
Abaqus-FRANC3D fracture mechanics sub-model with inserted surface crack.

**Figure 11:**
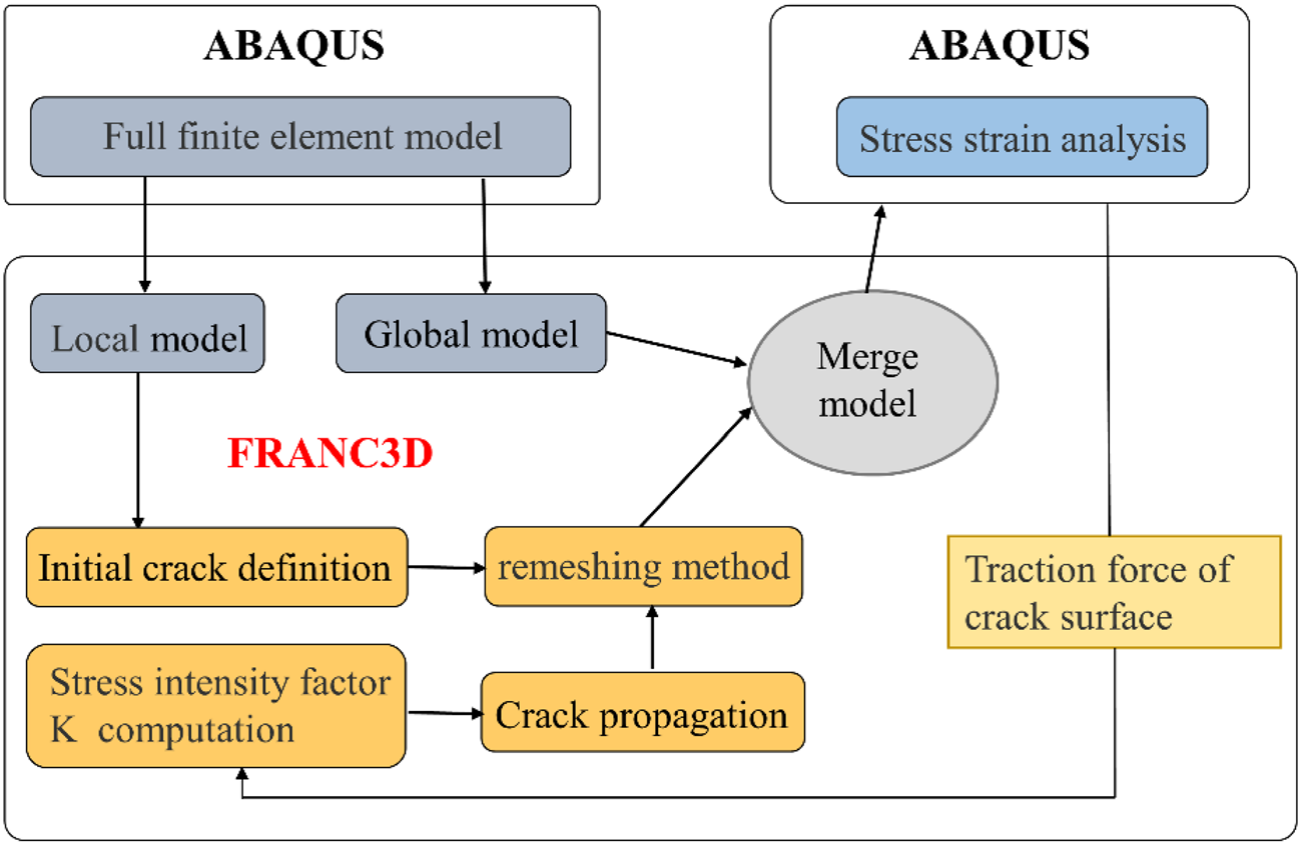
The co-simulation process of Abaqus and FRANC3D.

Initial fracture size (IFS) is the minimum crack length that can be detected using different techniques such as visual inspection, X-ray photography, ultrasonic reflection, and current applications. For engineering analysis, the IFS of the objects studied in this article is generally considered to be 0.5 mm. After the initial micro-crack is introduced, the grid near the crack is refined by the FRANC3D grid algorithm to ensure calculation accuracy, as shown in Fig. 10. The stress result under the action of preload was introduced into FRANC3D, and an axial alternating cyclic load was applied on clamp1 to realize the stress superposition on the crack surface. The fatigue crack propagation simulation was realized by using the median crack propagation technique to predict the fatigue crack propagation life. The co-simulation process of Abaqus and FRANC3D is shown in Fig. 11.

### The validation of the simulation method

#### Experimental results

**Figure 12:**
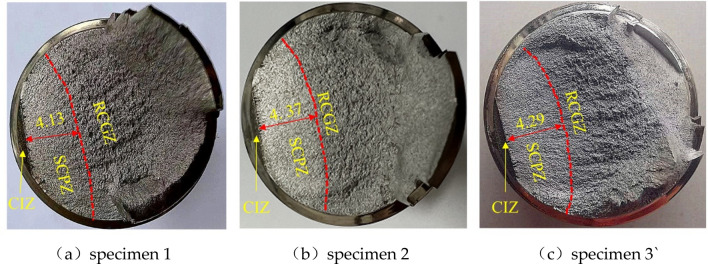
Fatigue fracture morphologies of bolts.

The fatigue fracture surfaces of the bolt specimens were obtained following the fatigue test. The bolt fracture specimens of approximately 10 mm in length were obtained by wire-cut electrical discharge machining. To demonstrate the fatigue failure mechanism, the fracture macroscopic morphologies of high-strength bolt specimens were examined using an optical microscope, as shown in Fig. 12. Using the 2D measuring program DS-300 in OM, the crack propagation depth can be precisely quantified^39^. The fatigue data obtained from the tests are shown in Table 2, where the fatigue life refers to the number of load cycles in which the bolt propagates from the prefabricated micro-crack to the complete fracture.

**Table 2:** Fatigue test results.

Specimen	Specimen 1	Specimen 2	Specimen 3
Fatigue life (N)	10,937	11,641	11,339
Depth of crack propagatio n (a/mm)	4.13	4.37	4.29

As can be seen from Fig. 12, due to the load ratio R = 0.3, the high-strength bolt was always in the tensile state under the coupling action of the pre-tightening force and the axial excitation load, and the normal stress is generated by the axial load. Therefore, the initial crack propagated steadily along the depth and side under the cyclic action of normal stress until the fracture. The fracture surface has obvious fatigue fracture characteristics, which can be separated into three different zones: the crack initiation zone (CIZ), the stable crack propagation zone (SCPZ), and the rapid crack growth zone (RCGZ). Before the fatigue test, the micro-crack was prefabricated by wire cutting at the maximum predicted stress of the thread bottom, so the prefabricated micro-crack area is regarded as CIZ. The zone SCPZ is a relatively smooth zone, in which the crack front appears semi-elliptical and extends all around, which is perpendicular to the direction of crack propagation, while the RCGZ zone is relatively rough. The central dimple zone and the marginal shear zone are typical shapes, which show the typical plastic failure characteristics. This means that when the length of the section crack reaches a certain threshold, the crack enters an unstable stage of rapid propagation, which eventually leads to the complete fracture of the bolt.

**Figure 13:**
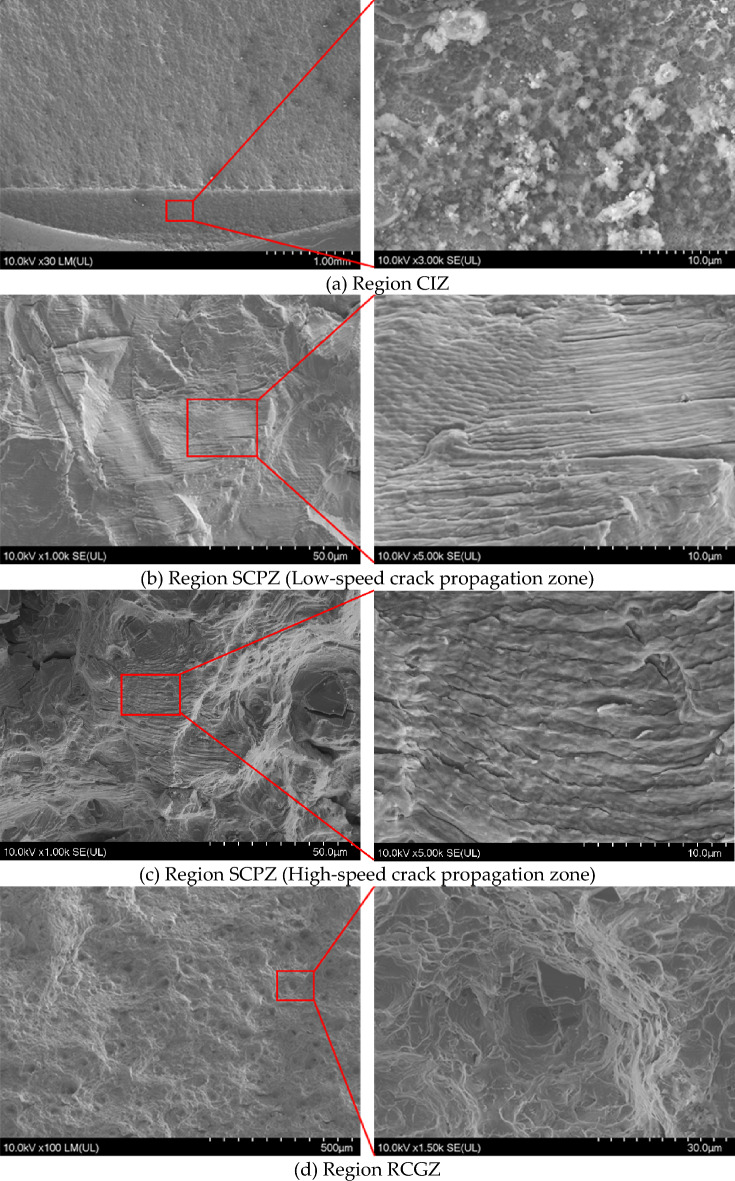
Microscopic fracture morphology of specimen 1.

To fully comprehend the three different regions of the fracture morphology, the fracture morphology of the bolt specimen was observed by scanning electron microscope (SEM), and the changes in the main fracture mechanism during crack propagation were demonstrated. The fracture morphology of specimen 1 is shown in Fig. 13. As shown in Fig. 13a, the particle residues in the area CIZ are mainly process residues and oxides produced by wire cutting during the prefabrication of micro-cracks. In Fig. 13b,c, the trans-granular fracture mechanism is observed to dominate, and the fracture surface undulation characterized by fatigue fringes is formed clearly. Among them, Fig. 13b is the low-speed growth zone of the initial section, the crack propagation rate is relatively slow, and the fracture surface is smoother than in the later stage of the crack propagation. As the number of fatigue cycles increases, the fatigue crack propagation rate increases, and the fracture surface becomes rough, as shown in Fig. 13c. The underlying cause is that as radial crack depth increases, the energy release rate of the crack tip also increases. This causes an exacerbation in the micro-mechanical activity of the fatigue crack tip and an increase in the roughness of the fracture surface. It is shown that the stress intensity factor at the crack tip is positively correlated with the depth of the crack tip. As shown in Fig. 13d, there are a lot of loose pits in the typical fast fracture morphology. Due to the large preload, the friction and wear of the shear stress are restrained to some extent by the normal stress, and the dimples in the instantaneous fracture zone are large, deep, and irregular, which are typical equiaxed dimples.

#### Simulation results

(1) Stress intensity factor (SIF)

**Figure 14:**
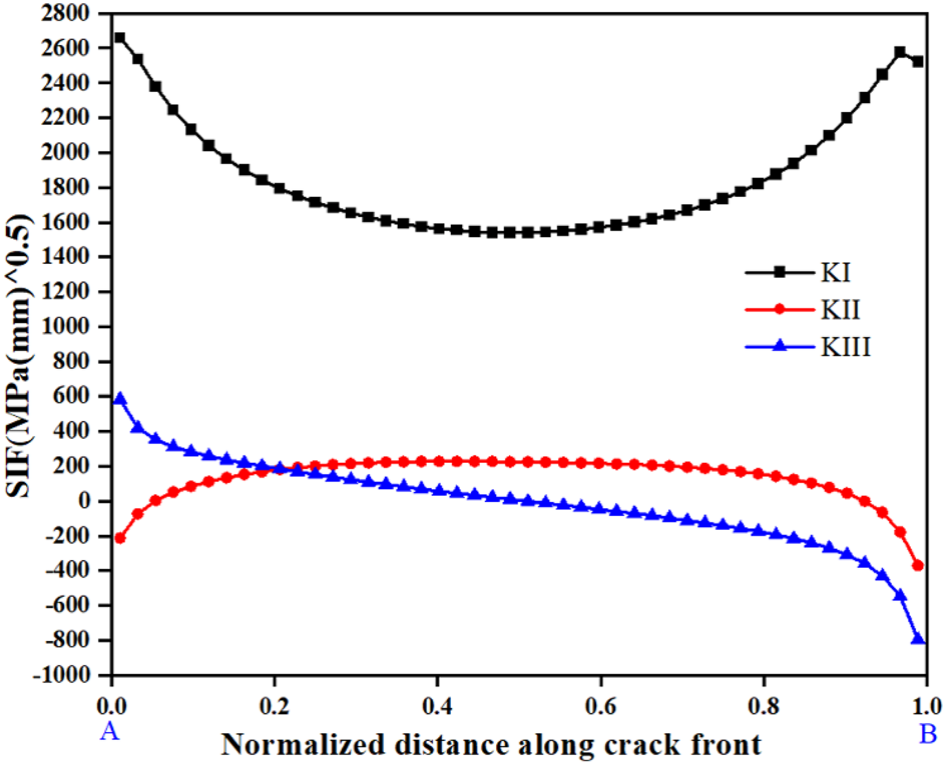
The stress intensity factor of the initial crack.

Since the three-dimensional crack front is usually irregular, the crack length is variable. Therefore, it is necessary to normalize the size of the crack. The normalized distance of the crack is taken as the ratio of the arc length from a point on the crack front to the starting point over the entire arc length. A semi-circular crack with an initial size of 0.5 mm was introduced at the location of maximum stress, and the SIF of all nodes at the crack front was calculated based on the M-integral, as shown in Fig. 14, the horizontal coordinate is the normalized crack length, and the vertical coordinate is the stress intensity factor. Take R = 0.3 as an example, from Fig. 14, it is evident that the $K_{I}$ of the crack front is between 1539 ~ 2657 ${\text{MPa}}\sqrt {{\text{mm}}}$, while $K_{II}$ and $K_{III}$ are between − 371 ~ 228 ${\text{MPa}}\sqrt {{\text{mm}}}$ and − 796 ~ 584 ${\text{MPa}}\sqrt {{\text{mm}}}$ , respectively. Since $K_{I}$ is significantly larger than $K_{II}$ and $K_{III}$, the fatigue crack is mainly propagated by model I in the current case, and the distribution rule is small in the middle and large at both ends. According to Eq. (1), the $K_{I}$ equates to the $K_{equiv}$ approximately, which is compared to the $K_{C}$ to assess whether or not the rapid instability stage is reached.

**Figure 15:**
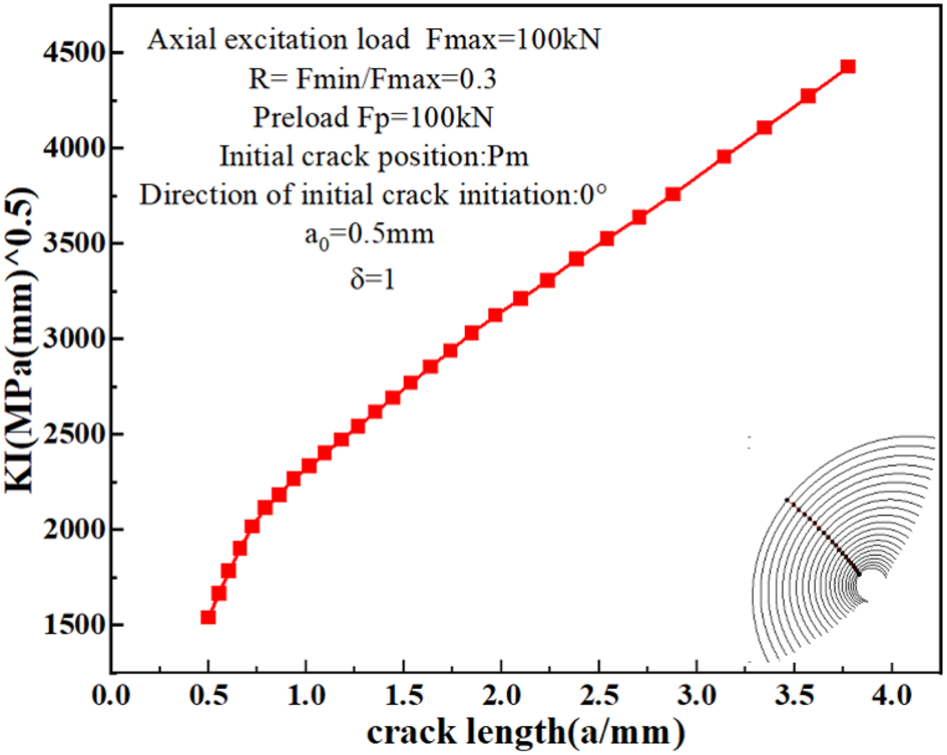
The variation of the maximum SIF along the crack depth.

As the crack continues to propagate, the SIF of the crack front increases. Figure 15 shows the variation of the maximum SIF along the crack depth. It can be seen that when the crack length increases to a certain value, the tested bolt cannot satisfy the condition of continuous crack growth, and fatigue failure occurs.

(2) Crack propagation

**Figure 16:**
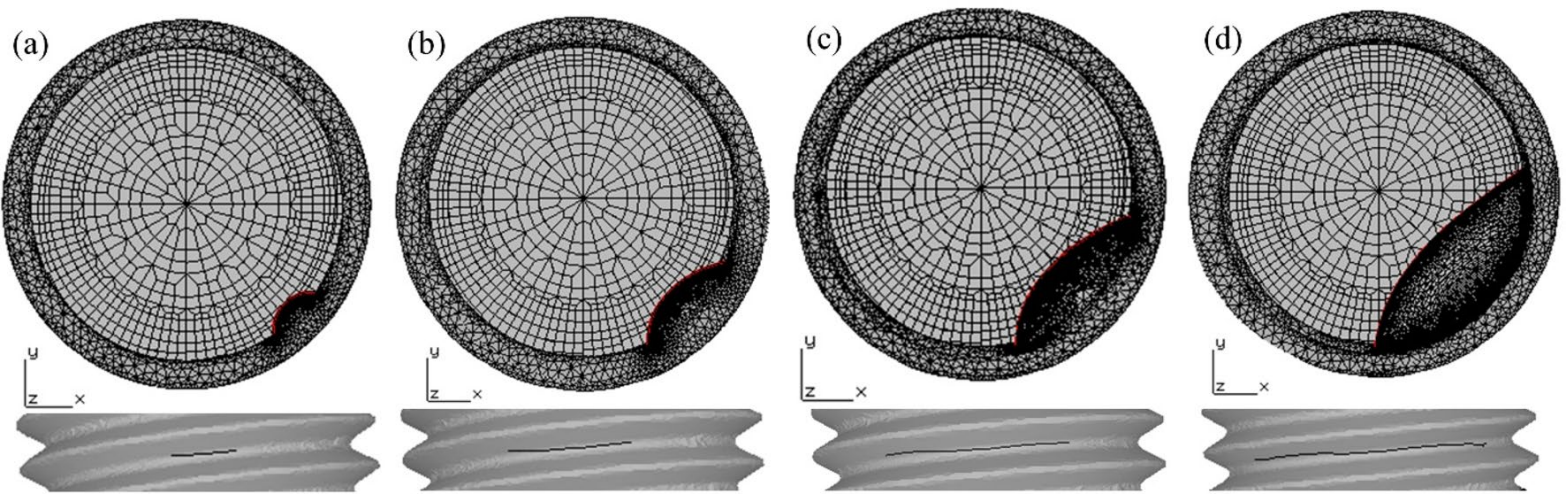
The crack propagation path on the thread bottom: (**a**) N = 2467; (**b**) N = 5907; (**c**) N = 8147; (**d**) N = 10,510.

Figure 16 shows the propagation history of fatigue crack length with the number of cycles under the coupling action of preload and axial cyclic load. The crack surface gradually evolves from the half circle to a similar half ellipse. Figure 16c,d show that as the crack propagates, the fracture cross-section area increases rapidly with fewer cycles, indicating that the crack has reached the rapid propagation stage.

It is found that the crack propagation path of a high-strength bolt simulated by FRANC3D under the same conditions is completely consistent with the fracture morphology of the bolts obtained by the experiment. At the same time, the simulated critical crack radial depth of the bolt fracture section is a = 3.92 mm, and the fatigue life is N = 10,510. Compared with the experimental results shown in Table 2, the error of the crack propagation critical depth is 5.1%, 10.3%, and 8.4%, respectively, and the error of fatigue life is 3.9%, 9.7%, and 7.3%, respectively. The experimental outcomes are in good agreement with the simulation results, which verify the accuracy of the simulation analysis of high-strength bolt crack propagation using FRANC3D.

Therefore, it is considered accurate to use FRANC3D to simulate the fatigue crack propagation of high-strength bolts using the same model and setting. Based on this, the impacts of the geometrical factors of the initial crack (e.g., position, direction, size), load factors (e.g., load ratio, mean load, load range), bolt preload, and friction coefficient of the screw pair on the fatigue crack propagation behavior of bolts were further investigated.

## Analysis of the influencing factors on crack propagation behavior

### Initial crack state

#### Crack initiation location

**Figure 17:**
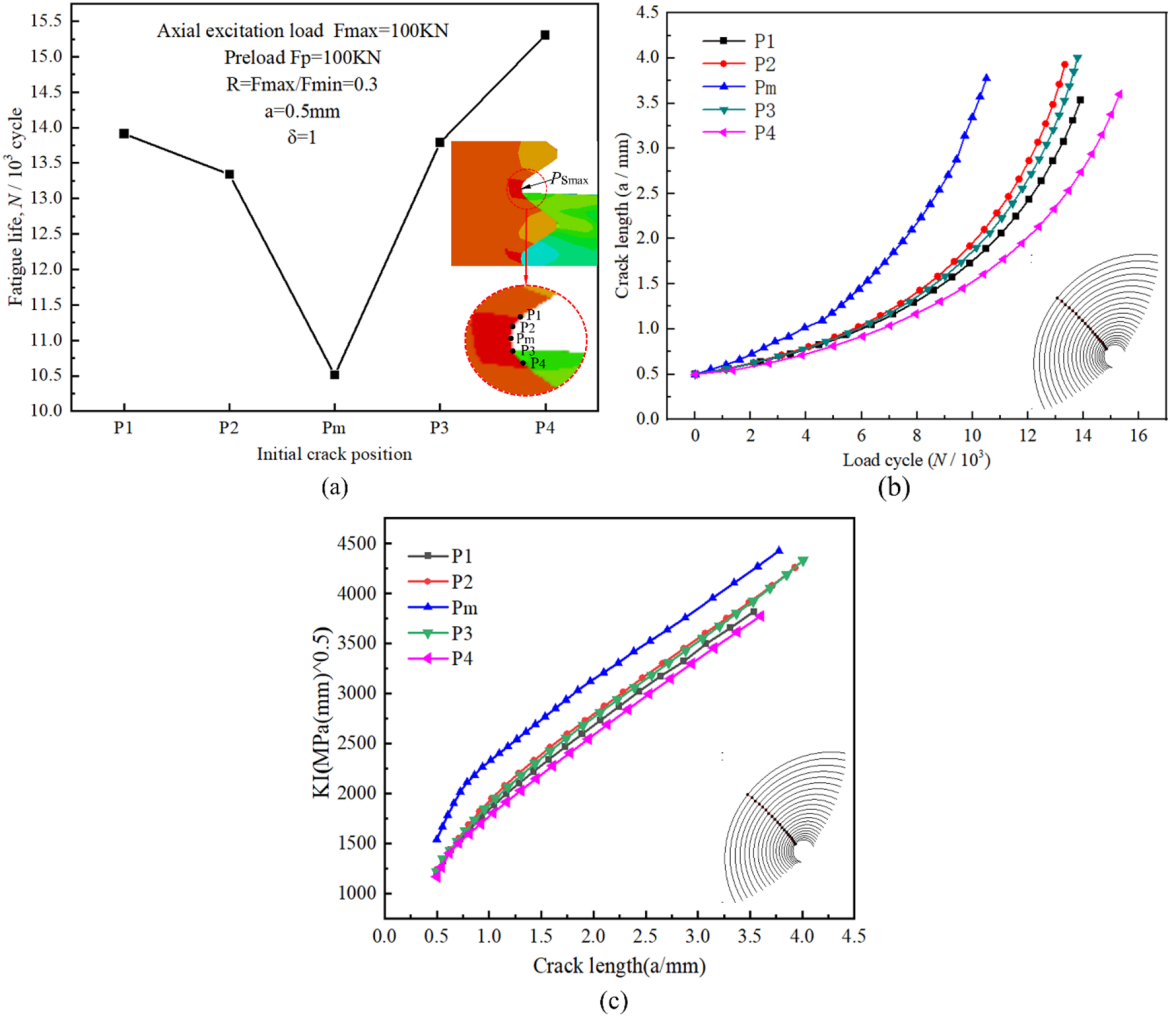
(**a**) Fatigue crack propagation lives versus the positions of the initial crack at the thread bottom; (**b**) Crack propagation process for various initial crack locations at the thread bottom; (**c**) The variation of the stress intensity factor along the crack depth at different initial positions.

The fatigue cracks typically occur at the maximum stress areas of the threaded bottom, but the initial locations of the fatigue cracks vary with different manufacturing processes and loading conditions. To evaluate the impact of the initial crack location on the fatigue crack propagation behavior, the initial micro-cracks were preset at several adjacent different locations in the first engaged tooth of the thread, including the maximum principal stress position (Pm) and four other adjacent positions (P1-P4) along the thread base. The initial length a_0_, size coefficient δ, locations, friction coefficient f of the thread pair, and loading conditions of all initial cracks are shown in Fig. 17a, and the grid and boundary conditions are the same as above. The fatigue life predicted by FRANC3D is contrasted in Fig. 17a. Obviously, the crack propagation life at Pm is the shortest predicted by numerical simulation, and the lives at other locations are greater than that at Pm and 1.27 to 1.46 times the lifetime of Pm. As a result, the crack propagation path is affected by the crack initiation position, which in turn affects the crack propagation life.

To further assess the rate of crack propagation at all crack initiation locations, Fig. 17b shows the relationship between the propagation depth of the same midpoint of the crack front and the number of load cycles for the initial cracks at different positions. It can be seen from the diagram that the crack propagation rate is slow at first and then fast. When the crack front SIF gets close to the fracture critical value of the material, the fatigue crack begins to extend rapidly and become unstable, and the crack initiation positions also affect the growth rate. The crack propagation rate at Pm is higher, while the rest of the crack initiations are relatively slow due to the stress intensity factor value ΔK of the initial crack. Of all the cracks, the one produced at P4, far from the thread bottom, is the safest.

To further evaluate the variation pattern of the stress intensity factor (SIF) with crack propagation, as shown in Fig. 17c, the SIF at the same midpoint of the crack front but with different initial positions was taken for characterization. From the figure, it can be observed that the SIFs of cracks at different positions exhibit the same variation trend with crack propagation depth, which exhibits a rapid increase from the initial non-linearity to the subsequent linearity. This overall trend is consistent with the conclusion that fatigue cracks propagate at a slow-fast rate. Additionally, the SIF increases the fastest at position Pm, which is consistent with the shortest lifespan at position Pm.

#### Crack initiation orientation

**Figure 18:**
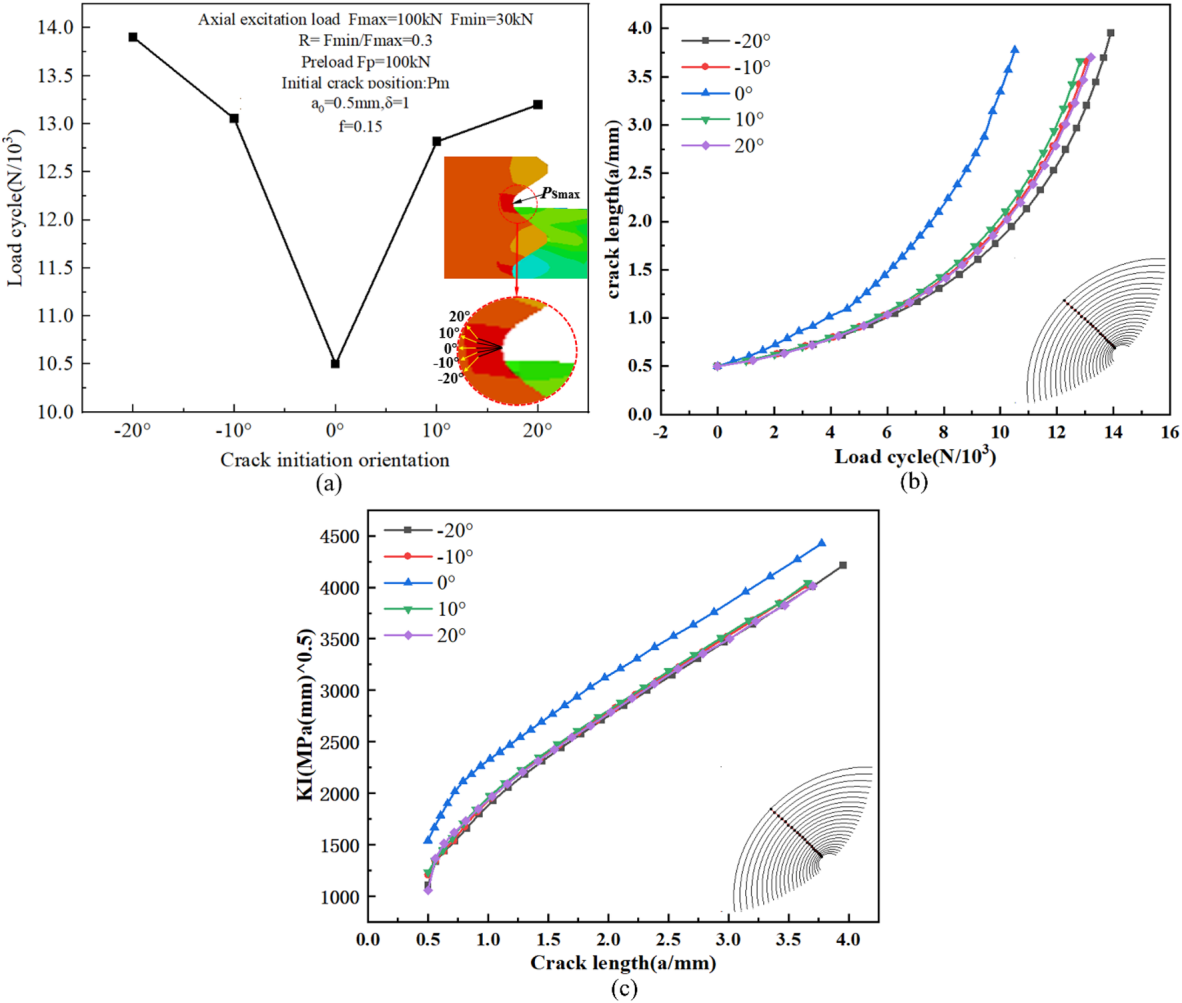
(**a**) Fatigue crack propagation lives versus the different orientations of the initial crack at thread bottom; (**b**) Crack propagation process for various orientations of initial crack at Pm; (**c**) The variation of the stress intensity factor along the crack depth in different orientations.

The direction of the initial crack surface is related to the loading conditions of the bolt and the local properties of the material. In order to evaluate the impact of the initial crack direction on the fatigue crack propagation behavior, different directions of initial cracks were pre-set at point Pm, including 0° (perpendicular to the axis of the bolt), 10°, 20°, − 10°, and − 20°. As shown in Fig. 18a, the initial crack size, load, mesh generation, and boundary conditions were the same as above. Obviously, the numerically simulated fatigue crack propagation life is the shortest in the 0° direction, and the fatigue life of the other four directions is 1.22 ~ 1.32 times longer than that of the 0° direction.

To further assess the propagation rate of the initial crack in various directions, Fig. 18b presents the relationship between the radial crack propagation depth and the number of load cycles for different initial crack directions. It can be observed from the graph that the crack propagation rate is also initially slow and then becomes faster. When the stress intensity factor at the crack front approaches the fracture critical value of the material, the fatigue crack begins to propagate rapidly and becomes unstable. and the crack initiation direction also affects the propagation rate. The crack propagation rate is higher in the 0° direction, while the rest of the crack directions are relatively slow due to the stress intensity factor value ΔK of the initial crack, which is consistent with the conclusion reached by Shakeri^40^ that cracks with a high inclination angle have less harm to fatigue performance compared to cracks with a smaller initial inclination angle. Among all the cracks, the most dangerous crack occurs in the 0° direction perpendicular to the bolt axis.

To further evaluate the variation of the stress intensity factor (SIF) of initial cracks in different directions with crack propagation, as shown in Fig. 18c, the SIF at the same midpoint of the crack front was taken for characterization. From the figure, it can be seen that the stress intensity factors of initial cracks in different directions also exhibit the same trend with increasing crack depth, showing a characteristic of a rapid increase from the initial non-linearity to the subsequent linearity. This is consistent with the conclusion that fatigue cracks propagate at a slow-to-fast rate. Additionally, the SIF increases fastest in the 0° direction, which is consistent with the shortest life in the 0° direction.

#### Initial crack length

The critical crack size is directly correlated with the fatigue crack propagation rate. From this perspective, the fatigue fracture behavior of high-strength bolts is significantly influenced by the initial crack length. To prove this viewpoint, six different initial crack lengths, including 0.2 mm, 0.5 mm, 0.8 mm, 1.0 mm, 1.5 mm, and 2.0 mm, were presupposed in the 0° crack direction at position Pm. The load, grid division, and boundary conditions were as previously described.

**Figure 19:**
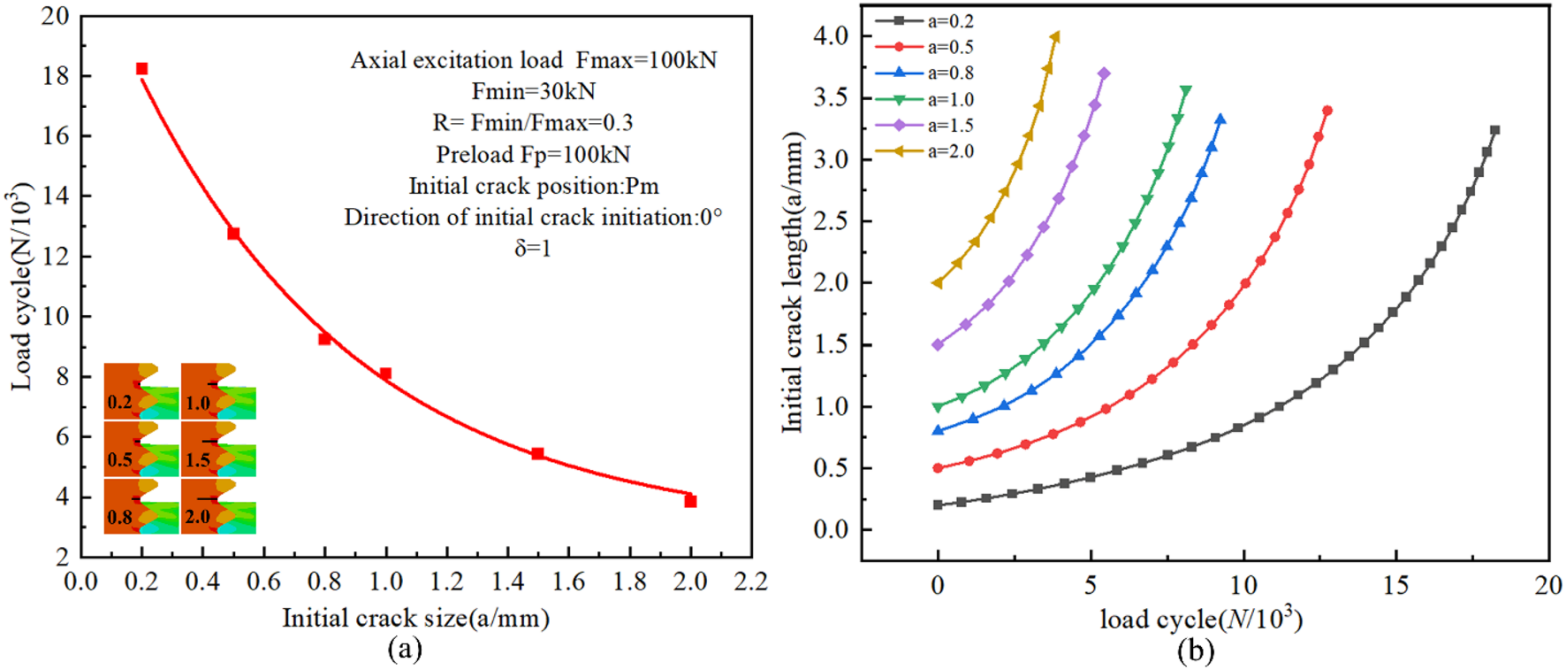
(**a**) Fatigue crack propagation lives versus the initial crack length at thread bottom; (**b**) Crack propagation process for various initial crack lengths at Pm.

A comparison of the crack propagation rates is shown in Fig. 19a. Clearly, the fatigue lifetime decreases as the initial crack length increases. When the initial crack length exceeds 1.5 mm, the fatigue life is reduced to only a few thousand cycles, and the stable crack propagation stage is not obvious. The fatigue cracks propagate rapidly until ultimate failure, which is basically consistent with the predicted critical lengths of 1.5 ~ 2mm for fatigue cracks in different directions at Pm. Figure 19b shows the fatigue lives of the bolts at different initial crack lengths. The overall exponential decay observed in the figure is as follows:

10 $$ {\text{N}} = 19.7728{\text{e}}^{{\left( { - \frac{a}{0.7365}} \right)}} + 2.7988 $$

#### Initial crack surface geometry

**Figure 20:**
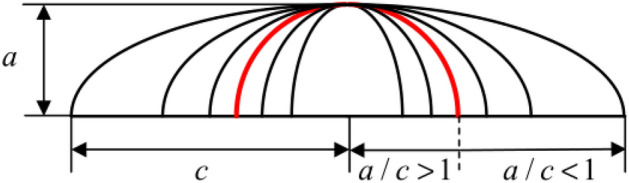
Initial crack surface geometry.

In order to evaluate the impact of the initial crack surface geometry on the fatigue crack propagation behavior of high-strength bolts, as shown in Fig. 20, the ratio of length to diameter of the initial crack is defined as the coefficient δ, i.e., δ = a/c. By setting the initial crack at the 0° direction of the Pm position and keeping the crack depth (a = 0.5 mm) constant, nine crack patterns with different δ values were introduced, namely 5/1, 5/2, 5/3, 5/4, 5/5, 5/6, 5/7, 5/8, and 5/9. The load, grid division, and boundary conditions were the same as above.

**Figure 21:**
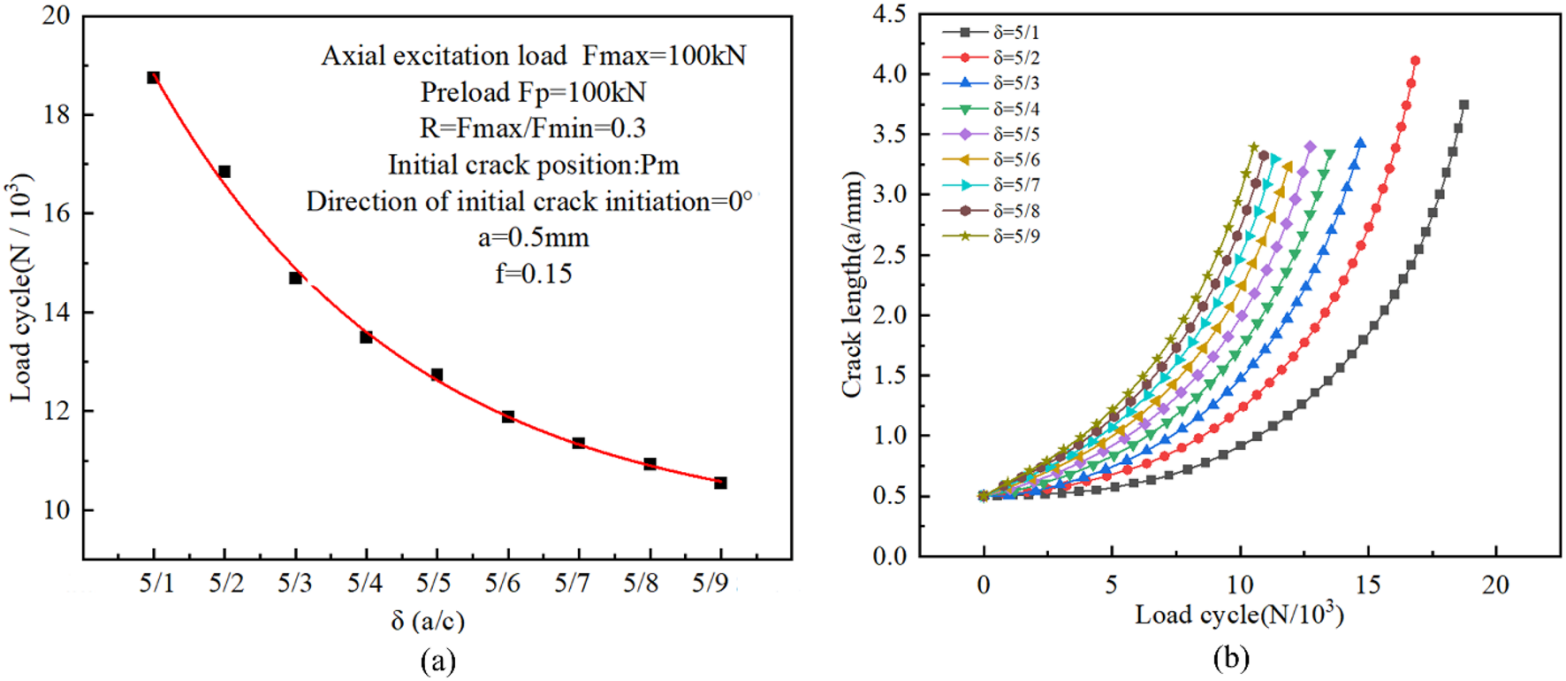
(**a**) Fatigue crack propagation lives versus initial crack surface geometries at thread bottom; (**b**) crack propagation process for various δ of initial crack at Pm.

The relationship between the predicted crack propagation rate, residual fatigue life, and the initial crack surface geometry is shown in Fig. 21. The results demonstrate that as the initial crack length c increases, the fatigue life of the bolt decreases with the severity of the initial crack defect.

The relationship between fatigue crack propagation life and crack propagation length can be used to qualitatively analyze the impact of initial crack geometry on fatigue behavior. Figure 21b shows that, at the early stage of crack propagation, when the value of a is constant, the smaller the value of c is, the slower the propagation rate at a given crack depth is. Whereas at the later stage of crack propagation, the difference in the propagation rate of the crack is small with different c values, which is because the propagation crack size tends to be a stability factor.

As shown in Fig. 21a, when a/c > 1, the bolt is more susceptible to the length of the crack, and the crack propagation life decreases significantly with the increase of the c value, whereas when a/c < 1, the crack propagation life decreases slowly with the increase of the c value. The reason is that as the c value increases, the crack area approaches the critical value of fracture, the shape coefficient approaches a stable value, and the residual life varies little. The overall exponential decline observed is described below:

11 $$ {\text{N}} = 12.20527{\text{e}}^{{\left( { - \frac{2c}{{0.72213}}} \right)}} + 9.56868 $$

**Figure 22:**
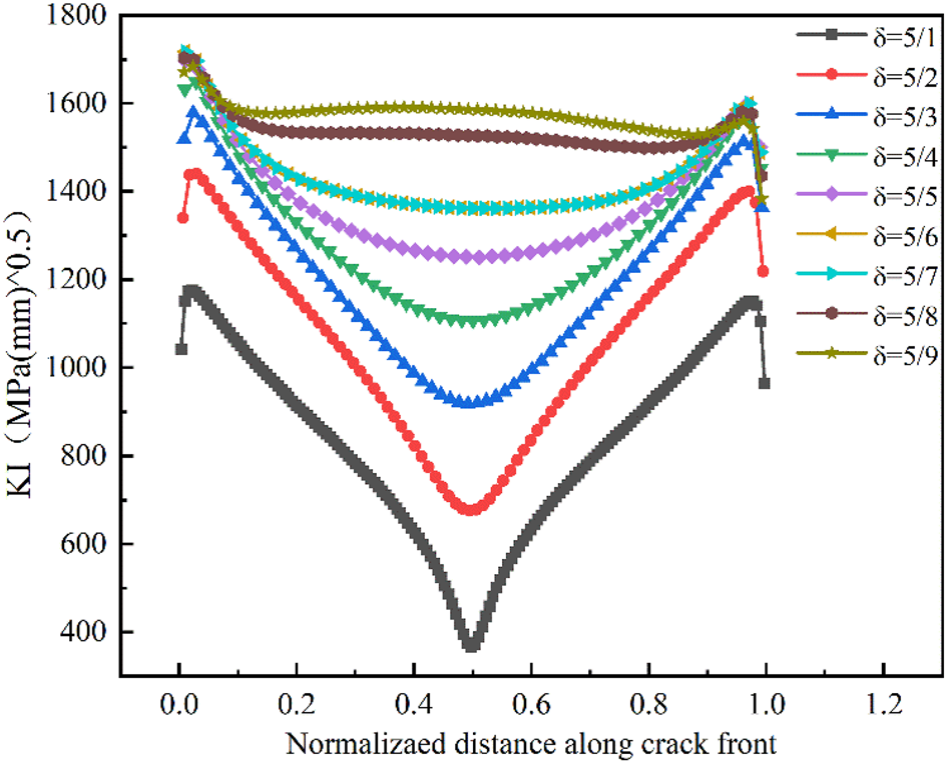
The stress intensity factor of crack fronts with different initial geometric shapes.

**Figure 23:**
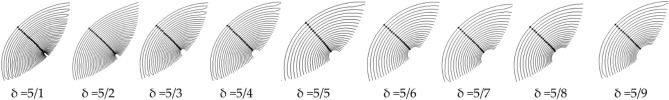
The evolution process of different crack shapes (a/c) after propagation.

To further evaluate the propagation evolution process of initial cracks with different shapes, combined with Figs. 22 and 23, it can be seen that the crack propagation was significantly affected by the shape ratio. When the crack is sharp (a/c > 1), the stress intensity factor at the crack front is large at both ends and small in the middle. As a result, circumferential propagation dominates the early stage of crack propagation. Whereas when the crack is gradually smooth (a/c < 1), the maximum points of the stress intensity factor at both ends move towards the middle of the crack front, Therefore, the crack mainly propagates along the radial direction.

### Cyclic loading condition

In Section "Initial crack state", the fatigue crack propagation behavior of bolts under a given cyclic load was investigated. Since axial load is the main factor in bolt fatigue, the effects of cyclic loads on crack propagation life will be discussed in this section. The cyclic load conditions were determined by a given maximum load Fmax or a given minimum load Fmin and the corresponding load ratio. The fatigue performance is evaluated through the R-N curve, which depicts the relationship between the load ratio and the number of cycles to failure.

In general, for a given Fmax, the mean load will increase with the increase in load ratio, which will shorten the fatigue life. In this paper, two schemes were used to carry out the comparative simulation, Scheme A: Fmax = 100kN was fixed, and the mean load was changed by gradually increasing the load ratio R, resulting in an increase in the mean load; Scheme B: Fmin = 30kN was fixed, and the mean load was changed by purposefully increasing the load ratio R, but this scheme resulted in a decrease in the mean load. The individual and combined influences of load ratio, mean load, and load range could be discussed simultaneously through these two simulation schemes.

**Figure 24:**
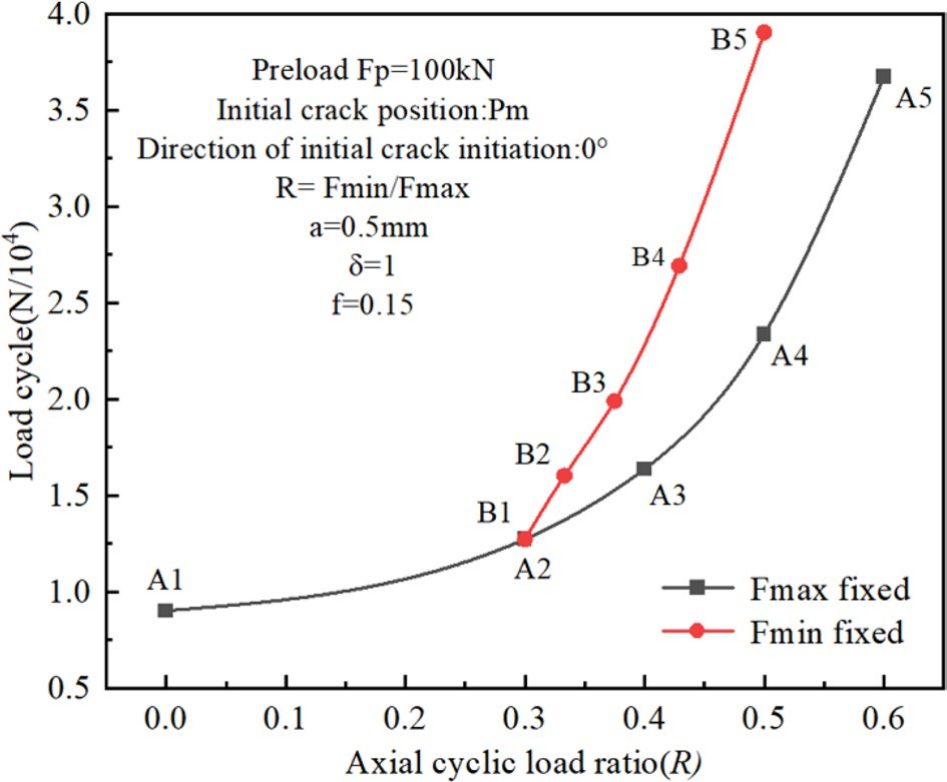
The influence of different axial cyclic load ratios on crack propagation life.

The relevant parameters of the initial crack are shown in Fig. 24. Boundary conditions and grid partitioning were also set as above. All simulation results are shown in Fig. 24 and Table 3.

**Table 3:** Simulation parameters for Fig. 21.

Load condition	R	Fmax/kN	Fmin/kN	Mean load/kN	Load range/kN
A1	0	100	0	50	100
A2	0.3	100	30	65	70
A3	0.4	100	40	70	60
A4	0.5	100	50	75	50
A5	0.6	100	60	80	40
B1	0.3	100	30	65	70
B2	0.333	90	30	60	60
B3	0.375	80	30	55	50
B4	0.429	70	30	50	40
B5	0.5	60	30	45	30

Figure 24 demonstrates that the fatigue performance of the bolts is significantly influenced by the load ratio R. Both schemes exhibit the same overall tendency, namely, the fatigue life gets longer as the load ratio rises. The reason is that the increase in load ratio reduces the SIF range (ΔK), and the fatigue crack propagation rate is also reduced.

As shown in Table 3, the comparison of A1 and B4 confirms that when the mean loads are the same, the smaller the load ratio is, the larger the load range is, and the shorter the fatigue life is. The comparisons of A3 and B2, A4 and B3, and A5 and B4 confirm that under the same load range, the fatigue life decreases with the increase in the mean load. Based on Fig. 24 and Table 3, we find that load ratio is not the only factor determining the fatigue life, the effects of cyclic loading conditions on high-strength bolt fatigue behavior are complex and nar. To evaluate fatigue life, factors such as load ratio, mean load, and load range must be taken into account comprehensively. Other loading factors, such as the working environment, bolt material, and processing technology, are also important, which are outside the purview of this research.

### The effect of preload

**Figure 25:**
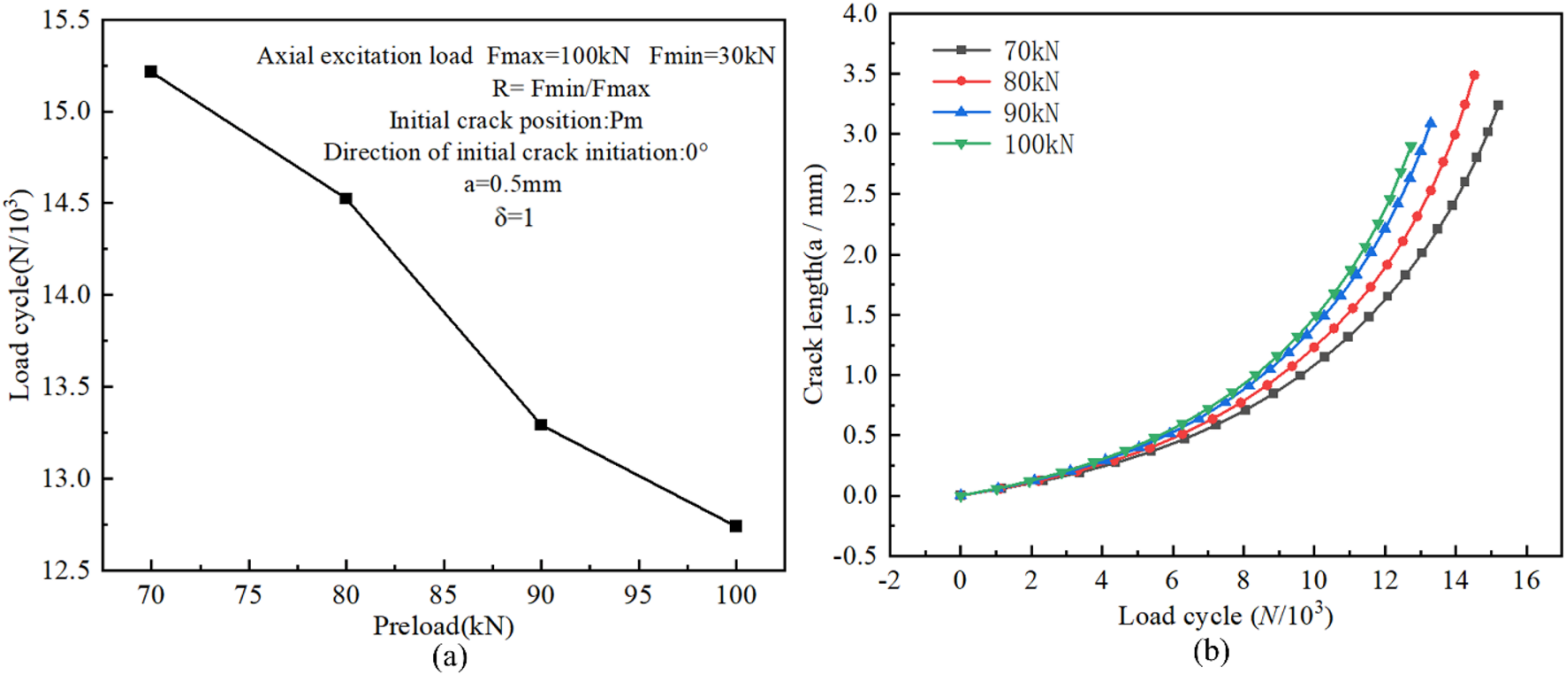
(**a**) Fatigue crack propagation lives versus the bolt preload; (**b**) Crack propagation process for various bolt preloads at Pm.

Proper bolt preload can effectively prevent the bolt from loosening under the action of an external load and prolong the fatigue life of the bolt. In order to evaluate the impacts of preload on the fatigue crack propagation behavior of the high-strength bolts, the pertinent settings of the initial crack were preset, as indicated in Fig. 25a, and four different bolt preloads were applied: 70kN, 80kN, 90kN, and 100kN, respectively. The boundary conditions and grid partitioning were also taken as above. All simulation results are shown in Fig. 25.

As can be seen from Fig. 25a, the fatigue crack propagation rate of the bolt is significantly influenced by the bolt preload. The greater the bolt preload, the shorter the crack propagation life. Figure 25b also shows that the smaller the bolt preload, the smoother the initial stage of crack propagation and the relatively longer the stable propagation life. The reason for this is that the SIF range (ΔK) of the crack front is increased by a larger preload, and the fatigue crack propagation rate also increases as a result. Thus, within a certain range, a larger bolt preload can effectively prevent bolt loosening and prolong the crack initiation life. However, once the initial crack is initiated, a larger bolt preload will reduce the crack propagation life.

### The influence of friction coefficient

The initiation life of a fatigue crack in a bolt is sensitive to the friction coefficient of the screw pair, and a reasonable combination of the friction coefficient and the bolt preload can significantly prolong the initiation life of the crack. It is necessary to discuss whether the friction coefficient also affects the crack propagation life.

**Figure 26:**
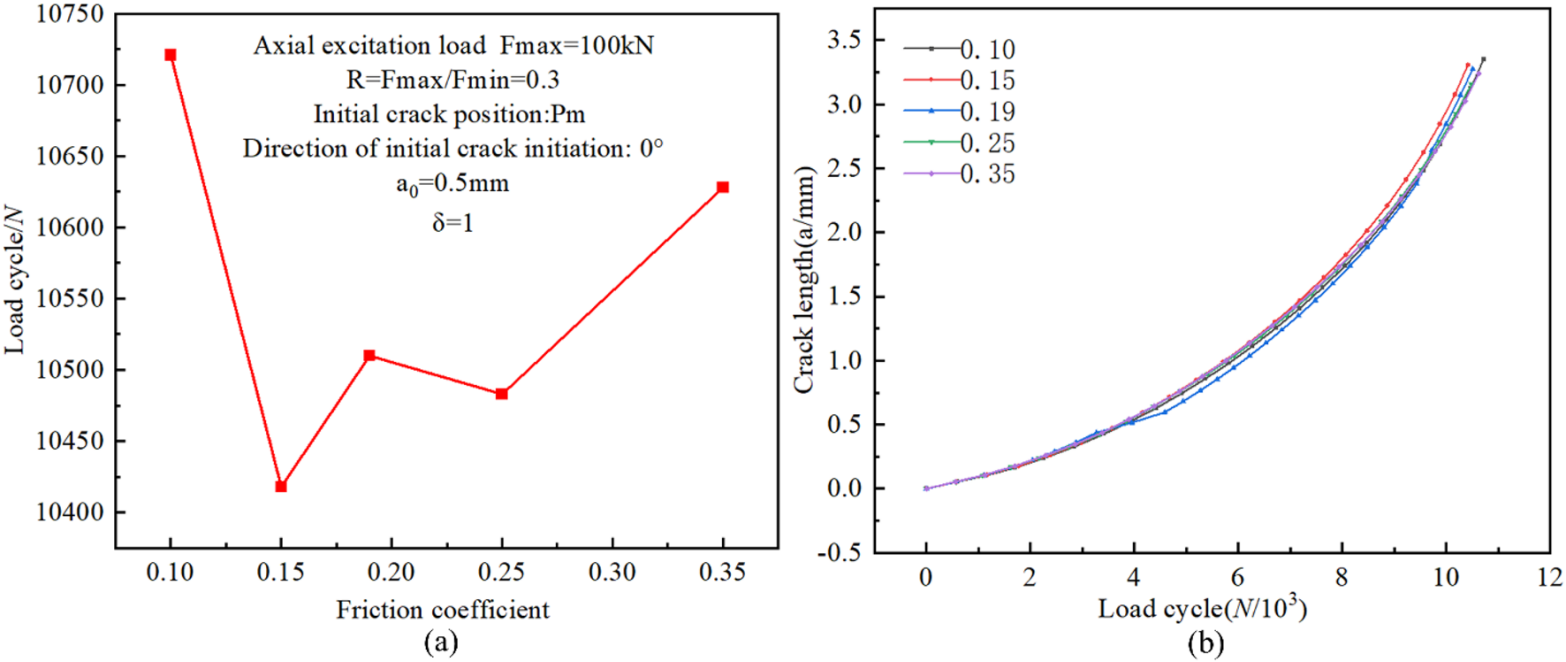
(**a**) Fatigue crack propagation lives versus friction coefficients of screw pair; (**b**) Crack propagation process for various friction coefficients of screw pair.

Similarly, the pertinent settings of the initial crack were preset, as indicated in Fig. 26a, five different friction coefficients were selected as 0.10, 0.15, 0.19, 0.25, and 0.35, respectively, which essentially covered the scope of engineering friction coefficients for the screw pair. The boundary conditions and grid partitioning were also taken as the same as above. All simulation results are shown in Fig. 26.

As can be seen from Fig. 26a, the difference in the fatigue crack propagation life of high-strength bolts is only within 300 load cycles under the five different friction coefficients. In addition, starting from Fig. 26b, there is almost no difference in the fatigue propagation rate and the critical length of bolt crack propagation with different friction coefficients of the thread pairs. This is because when there is an initial crack in the bolt, the main factor affecting the bolt fatigue life is the SIF of the crack front, the friction coefficient of the threaded pair has no obvious effect on the crack propagation life, which is consistent with the finding of Shakeri^40^ that the friction coefficient between the screw-nut pairs has a small effect on the stress intensity factor.

## Conclusions

In this research, the fatigue crack propagation behavior of high-strength bolts with initial crack defects is studied by experimental and numerical simulation. The main conclusions are as follows:

1. The stress state of a high-strength bolt under torque preloading force is obtained through finite element calculation, and the maximum stress is concentrated at the bottom of the first engaged thread, which is the point of fatigue crack initiation.
2. The calculation results of the anisotropic stress intensity factor by FRANC3D indicate that the mode I fatigue crack is the main bolt propagation under the combined action of preloading force and axial excitation.
3. The crack initiation location and crack direction have varying degrees of effect on the fatigue crack propagation life. When the initial crack length is preset at position Pm with an orientation of 0°, the fatigue extension life is the shortest, and the bolt connection is the most dangerous.
4. The fatigue crack propagation life is sensitive to the initial crack radial depth, the crack size coefficient, the axial excitation load, and the preloading force, but insensitive to the friction coefficient of the screw pair.

## Data Availability

All data generated or analyzed during this study are included in this article.
